# Surgical Management among Patients with Acetabular-Pelvis Fractures in a Trauma Care Centre

**DOI:** 10.31729/jnma.6492

**Published:** 2023-11-30

**Authors:** Yogendra Agrahari, Marie Joey Lambaco Agrahari, Sangita Karki Kunwor

**Affiliations:** 1 Department of Orthopaedics, Devdaha Medical College and Research Institute, Devdaha, Rupandehi, Nepal; 2 Hospice Department, Access Care Management Consultancy, Van Nuys, California, United States of America; 3 Department of Global Health and Development, Graduate School of Hanyang University, Seoul, South Korea

**Keywords:** *acetabulum*, *fracture fixation*, *pelvis*

## Abstract

**Introduction:**

Surgical management of pelvic and acetabular fractures due to high-energy trauma is one of the most challenging in orthopaedics. Most patients are often associated with other life-threatening injuries. Several studies demonstrated that accurate fracture reduction decreases the incidence of post-traumatic arthritis and improves functional outcomes. The aim of the study was to find out the prevalence of the surgical management among patients with acetabular-pelvis fractures in a trauma care centre.

**Methods:**

This is a descriptive observational study conducted at a trauma hospital from 1 September 2016 to 31 August 2020. Ethical approval was obtained from the Institutional Review Committee. Patients with displaced fractures of the pelvis ring or acetabulum were included in the study whereas isolated pubic rami fractures and pathological fractures were excluded from the study. Operative plans were decided after radiographic X-rays and 3-dimensional reconstruction computed tomography scan evaluation. A convenience sampling method was used. The point estimate was calculated at a 95% Confidence Interval.

**Results:**

Among 136 patients with acetabular-pelvis fractures, 64 (47.06%) (38.67-55.45, 95% Confidence Interval) underwent surgical management. The average time duration from injury to surgery was 7 days. All patients were able to weight bear 3 months.

**Conclusions:**

The prevalence of surgical management among patients with pelvic-acetabular fracture was found to be similar to the other studies done in similar settings.

## INTRODUCTION

Pelvic fractures account for approximately 3% of all fractures, usually the result of high-energy trauma and may have associated soft tissue and organ damage resulting in significant morbidity and mortality in these patients.^1^ Pelvic ring injuries and acetabular fractures, or a combination of both a distinctive challenges which are often displaced to such an extent that surgery is the inevitable treatment method and which reduces mortality and morbidity if early stabilization can be done.^2-4^

In 1964, surgical treatment evolved as a choice for restoration of joint congruity which is of paramount importance in reducing the incidence of early hip osteoarthritis and pain.^5,6^ The outcome of the fixation is dependent on many variables such as energy level of the injury, radiographic fracture pattern, surgeon's knowledge of pelvic anatomy, timing of surgery and appropriate choice of surgical approach.^7^

The aim of the study was to find out the prevalence of the surgical management among patients with acetabular-pelvis fractures in a trauma care centre.

## METHODS

This descriptive cross-sectional study was conducted at the Shree Tinau International Hospital, Butwal, Nepal from 1 September 2016 to 31 August 2020. Ethical approval was obtained from the Institutional Review Committee (Reference number: 020/2020). The patients with sacroiliac joint dissociation, acetabular protrusion, displaced fractures of the pelvic ring or acetabulum or both secondary to trauma were included in this study. Isolated undisplaced superior or inferior ramus or both rami fractures, isolated undisplaced or minimally displaced iliac wing, patients with co-morbidities, undisplaced isolated acetabular fracture and pathological fractures that could be managed conservatively were excluded from this study. Convenience sampling method was used. The sample size was calculated using the following formula:


$$\begin{array}{ll} \text{n} & ={\text{Z}}^{2}\times \frac{\text{p}\times \text{q}}{{\text{e}}^{\text{2}}}\\ & =1.96^{2}\times \frac{0.50\times 0.50}{0.09^{2}}\\ & =119 \end{array}$$

Where,

n = minimum required sample size
Z = 1.96 at 95% Confidence Interval (CI)
p = prevalence taken as 50% for maximum sample calculation
q = 1-p
e = margin of error, 9%

The calculated sample size was 119. However, 136 patients were included in the study.

All the fractures were managed initially following the standard multi-trauma and polytrauma management protocols. Patients with isolated acetabulum and pelvis injuries were directly admitted to the orthopaedic department. However, patients with multiple injuries example polytrauma were admitted to the intensive care unit with a multi-disciplinary department approach. Fractures were diagnosed using standard pre-operative radiographs: anteroposterior pelvis, inlet and outlet views of the pelvis and Judet view and computed tomography (CT) with 3-D reconstruction were advised. Clinical preoperative stabilisation of patients was ensured by the anaesthetists' team. Preoperative factors were studied and included the patient's age and gender, mechanism of injury, associated injuries and time to surgical intervention. Postoperative factors review includes the duration of hospital stay, complications and ambulatory status at the most recent follow-up. A written consent from the patient was taken and planned for surgery once he/she was hemodynamically stable. All the patients were given Cefazolin as preoperative antibiotics and antithrombotic stockings were applied and administered with low molecular weight heparin for prophylaxis against deep vein thrombosis (DVT). Sutures were removed 2 weeks post-operatively. Early mobilization was stressed, and patients were encouraged to sit up within the first 24-48 hours post-surgery and mobilization with toe touch weight bearing for 8 weeks was advised. Weight-bearing was progressively increased to full weight after 8 weeks while a 12-week delay was considered for patients with osteoporotic bone or comminuted fractures.

Data were entered and analysis was performed using IBM SPSS Statistics version 25. The point estimate was calculated at a 95% CI.

## RESULTS

**Figure 1:**
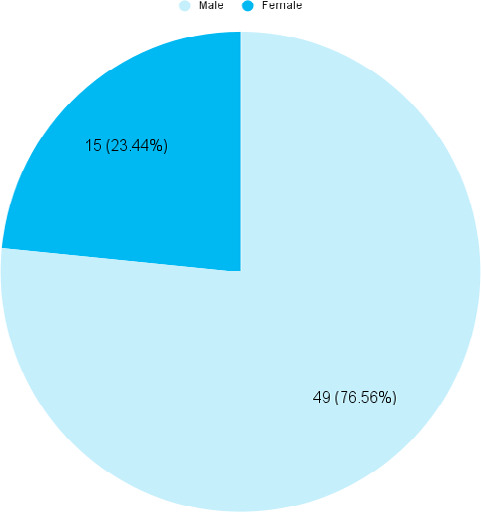
Gender-wise distribution (n= 64).

Among 136 acetabular-pelvis fractures, 64 (47.06%) (38.67-55.45, 95% CI) patients underwent surgical management. the rest had undergone conservative management. The mean age of patients was 38.63+4.29 years. Among them, male were 49 (76.56%) (Figure 1).

The mean duration of hospital stay was 11.5+4.30 days. Road traffic accident 47 (73.44%) is the common cause of injury, followed by an alleged fall 17 (26.56%). The average time duration from injury to surgery was 7 days. The patients who were able to weight bear 3 months post-operatively were 58 (90.63%). Tingling sensations were seen among 23 (35.94%) over the lower extremity which lasted for 6 months post-op follow-up.

## DISCUSSION

Among patients with acetabular-pelvis fractures 47.06% underwent surgical management. Around 3-4% of all fractures are usually associated with significant trauma of pelvic and acetabulum fractures and their management is one of the most challenging tasks in orthopaedic trauma which was similar to the findings study done by The Nepal Surgeons Overseas Assessment of Surgical Need (SOSAS).

This study has made the first countrywide population-based assessment regarding fall injuries, road traffic injuries and burn injuries which were 37.5%, 19.8% and 14.2% respectively.^8^ The prevalence of fall injury is higher than road traffic injuries for acetabular pelvis injury. These fractures often present in the context of polytrauma and may cause life-threatening hemodynamic instability. These patients should be managed by the trauma surgeon in an aggressive manner and early decision-making concerning the operative and non-operative management options for better postoperative outcomes.^9^

Early definitive stabilization of pelvic and acetabular fractures is optimal as it facilitates early functional rehabilitation. However, this is often not possible as patients often have various physiologic insults that require stabilization prior to treating the fracture.^2,10^ Timing for surgery has been shown to be very important as several studies reported poor results when open reduction and internal fixation were performed more than three weeks post-injury. In 1994 their study regarding poor clinical results in delayed reconstruction of acetabular fractures of more than 21 days compared to early intervention.^12^ In our study, the average time from the injury to surgery was 7 days. Regarding the infection, avascular necrosis was shown at 6.7% and 16.7% respectively,^13^ there were no complications in our study. However, 23 out of 64 (35.93%) patients had tingling sensations over the extremities which could be due to the sciatic nerve contusion which was resolved 6 months of postoperative care. In several studies, it has been shown that 11.8% have sciatic nerve palsy in the form of foot drop but were resolved after 8-11 months post-operatively.^14^

The limitation of this study is the small number of patients which could be due to the rarity of the cases compared to other trauma conditions. A larger number of patient population could help us to identify and predict the prognosis of these fractures.

## CONCLUSIONS

The prevalence of surgical management done in acetabular-pelvis fractures was similar to the studies conducted nationally and internationally. Improved clinical outcomes are associated with younger age, fewer concomitant injuries, shorter time intervals to surgery and more closely approximated anatomical fracture reduction.
